# Effect of Prophylactic Vaccination with the Membrane-Bound Acid Phosphatase Gene of *Leishmania mexicana* in the Murine Model of Localized Cutaneous Leishmaniasis

**DOI:** 10.1155/2021/6624246

**Published:** 2021-04-09

**Authors:** María Angélica Burgos-Reyes, Lidia Baylón-Pacheco, Patricia Espíritu-Gordillo, Silvia Galindo-Gómez, Víctor Tsutsumi, José Luis Rosales-Encina

**Affiliations:** Departamento de Infectómica y Patogénesis Molecular, Centro de Investigación y de Estudios Avanzados del Instituto Politécnico Nacional, México City, Mexico

## Abstract

Leishmaniasis is a disease caused by an intracellular protozoan parasite of the genus Leishmania. Current treatments for leishmaniasis are long, toxic, and expensive and are not available in some endemic regions. Attempts to develop an effective vaccine are feasible, but no vaccine is in active clinical use. In this study, the LmxMBA gene of *Leishmania mexicana* was selected as a possible vaccine candidate using the reverse vaccinology approach, and the prophylactic effect generated by DNA vaccination with this gene in a murine model of cutaneous leishmaniasis was evaluated. The results showed that prophylactic vaccination with pVAX1::LmxMBA significantly reduced the size of the lesion and the parasitic load on the footpad, compared to the control groups. At a histological level, a smaller number of parasites were evident in the dermis, as well as the absence of connective tissue damage. Mice immunized with plasmid pVAX1::LmxMBA induced immunity characterized by an increase in the IgG2a/IgG1 > 1 ratio and a higher rate of lymphocyte proliferation. In this study, immunization with the plasmid promoted an improvement in the macroscopic and microscopic clinical manifestations of the experimental infection by *L. mexicana*, with a T helper 1 response characterized by an IgG2a/IgG1 > 1 ratio and high lymphoproliferative response. These findings support immunization with the plasmid pVAX1::LmxMBA as a preventive strategy against cutaneous infection of *L. mexicana*.

## 1. Introduction

Leishmaniasis is an endemic disease in 97 countries and is considered a neglected tropical disease. A total of 1 billion people living in endemic areas are at risk of acquiring the disease, and it is estimated that there are at least 12 million cases of the various forms of leishmaniasis worldwide [1–3]. Cutaneous leishmaniasis (CL) caused by *L. mexicana*, *L. amazonensis*, *L. major*, and *L. braziliensis* is the most common form of leishmaniasis [4]. Visceral leishmaniasis (VL) caused by *L. donovani* and *L. infantum* is the most severe and fatal disease [5].

The immune cell response is essential in the control of *Leishmania* infection. The development of specific T helper 1 (Th1) response, based on the production of proinflammatory cytokines, such as interferon-gamma (IFN-*γ*), interleukin 12 (IL-12), and tumor necrosis factor-alpha (TNF-*α*), can protect mammalian hosts from infection by the parasites. In contrast, a Th2 response supports the persistence of parasites and can cause an active disease [6, 7]. Over the past years, studies have been developed to identify new candidates for the prevention of leishmaniasis and generate protective immunity [8–10]. However, there is still no effective prophylactic vaccination against human disease. First-generation vaccines with attenuated parasites concern potential reversion to virulence and raise a risk of reversion to clinical disease in immunocompromised individuals. On the other hand, second-generation vaccines might induce mainly humoral responses and are poorly immunogenic when administered without the association of adjuvants [8, 11, 12].

Another approach is the use of antigen-encoding plasmid DNA as an efficient and practical method of antigen delivery and as induction of a protective immune response against various intracellular pathogens [13–15]. DNA vaccines have been used in experiments against human immunodeficiency virus, tuberculosis, Chagas disease, and leishmaniasis [16–23]. These plasmids contain cytosine-phosphate-guanosine (CpG) motifs, which bind and activate toll-like receptor 9 (TLR9), thus triggering the innate immune response and subsequent development of adaptative immunity, inducing the development of the Th1 response [24, 25]. In this context, this vaccination technology plays a promising role in the development of new candidates against CL, which requires Th1 cell immunity.

In the present work, the prophylactic potential of an intramuscular injection of plasmid DNA encoding the LmxMBA protein (membrane-bound acid phosphatase of *Leishmania mexicana*) was evaluated in BALB/c mice. Immunoinformatic tools selected the *L. mexicana* membrane-bound acid phosphatase (XP_003874608.1). LmxMBA protein was identified in the *L. mexicana* promastigote and amastigote stages by antibodies from mice immunized with recombinant protein. Due to the high conservation found in the amino acid sequence of this protein in different *Leishmania* species, the extramembrane region was cloned, purified, and evaluated as a DNA vaccine candidate in BALB/c mice against infection caused by this parasite. The vaccine efficacy was evaluated by measuring the size of the lesion in the footpad, the parasite load, and histopathological analysis of the lesion.

## 2. Materials and Methods

### 2.1. Cell Culture

*L. mexicana* promastigotes, strain MHOM/MX/92/UADY68, were axenically cultured at 28°C in RPMI-1640 medium (GIBCO), pH 7.4, supplemented with 10% fetal bovine serum and antibiotics (100 IU/ml penicillin and 50 *μ*g/ml streptomycin).

### 2.2. Animals

Female BALB/c mice (4-6 weeks old) were provided by the Animal Production and Experimentation Unit (UPEAL-CINVESTAV) following the specifications of the Mexican National Norm (NOM-062-ZOO-1999) that is a version of the *Guide for the Care and Use of Laboratory Animals* 2011. The research protocol (no. 0216-16) was approved by CINVESTAV's Institutional Animal Care and Use Committee (CINVESTAV-IACUC).

### 2.3. In Silico Analysis

*L. mexicana* annotated proteins from the TriTryp databases consisted of 8250 open reading frames (ORFs) (TriTrypDB-6.0_LmexicanaMHOMGT2001U1103_AnnotatedProteins). The ORFs were analyzed for the identification of signal peptides and transmembrane helices by TMHMM (http://www.cbs.dtu.dk/services/TMHMM/1) and TOPCONS (http://topcons.cbr.su.se/). The expression sequence tags (EST) and mass spectrometry (MS) data from the TriTryp site (https://tritrypdb.org/tritrypdb/) were examined to define the expressed transmembrane proteins. Consensus subcellular localization was determined by Euk-mPloc (http://www.csbio.sjtu.edu.cn/bioinf/euk-multi-2/), LocTree3 (https://rostlab.org/services/loctree3/), DeepLoc (http://www.cbs.dtu.dk/services/DeepLoc/), and CELLO (http://cello.life.nctu.edu.tw/). Fold change in expressed genes between promastigotes, axenic amastigotes, and macrophage-derived amastigotes was identified based on RNA Seq Evidence (Transcriptomic in https://tritrypdb.org/tritrypdb/).

### 2.4. Construction of Recombinant Plasmids

The eukaryotic expression vector pVAX1 (Invitrogen, Carlsbad, CA, USA) and the bacterial expression vector pCR4-TOPO (Invitrogen, Carlsbad, CA, USA) were chosen to clone and express the LmxMBA gene. Briefly, the 1337 bp DNA fragment containing the LmxMBA gene (GenBank No. XM_003874559.1, sequence positions 94–1431), lacking the transmembrane regions, was obtained by polymerase chain reaction (PCR) (Thermal Cycler spp., Thermo Fisher Scientific) using the genomic DNA of *L. mexicana* promastigotes as the template and the primers LmxMBA F *HindIII* (5′-AAGCTTTCGCCACCATGGACAAGGTGGAGCTGGTGCAG-3′) and LmxMBA/R *EcoRI* (5′-CACGAATTCTTACCCGCCGCTGGACATGGGCGAC-3′). The PCR reaction contained 1 *μ*g of template, 2 mM MgCl_2_, 0.2 mM of each dNTP, 1x PCR buffer, and 2.5 U of Taq DNA polymerase. PCR conditions involved initial denaturation at 94°C for 5 min, 35 cycles of primer annealing at of 94°C for 1 min, 65°C for 30 s, and 72°C for 1 min and a final extension at 68°C for 7 min. The amplified fragment was purified (QIAEX II, Invitrogen, Carlsbad, CA USA), cloned into pCR4-TOPO vector and subcloned into pVAX1. The recombinant plasmid pVAX1::LmxMBA was confirmed by double restriction enzyme digestion (*HindIII*/*EcoRI*) and DNA sequencing.

The prokaryotic expression vector pRSET A (Invitrogen, Carlsbad, CA, USA) was chosen to clone and express the recombinant protein LmxMBA. The primers used were LmxMBAP/F *BamHI* (5′-CGGATCCTACAAGGTGGAGCTGGTGCAGGTG-3′) and LmxMBAP/R *HindIII* (5′-GAAATATAAGCTTACCCGCCGCTGGACATGGGCGAC-3′). The content reaction and conditions of PCR reaction were previously described [26]. The amplified fragment was purified and inserted into the *BamHI* and *HindIII* restriction sites of pRSET A, obtaining the recombinant plasmid pRSETA::LmxMBA. The construct was sequenced to confirm the correct sequence of the gene after PCR and correct insertion of the gene in frame with the ATG of the plasmid.

### 2.5. DNA Purification

Plasmid pVAX1::LmxMBA was maintained and propagated in *E. coli* DH5. Endotoxin-free plasmid DNA was purified by anion-exchange chromatography using a PureLink™ HiPure Plasmid DNA Purification Kit (Invitrogen) according to instructions from the manufacturer, and DNA used for immunizations was resuspended in PBS pH 7.4. After purification, plasmid concentration was determined by absorbance at 260 nm and 280 nm. The OD 260/280 ratios for purified DNA were 1.8–2.0, indicating that preparations were free of major protein contamination.

### 2.6. Purification Recombinant Protein

To obtain the recombinant protein LmxMBA, *E. coli* BL21 pLysS cells were transformed with the recombinant plasmid pRSETA::LmxMBA and grown in Luria Bertani medium to an optical density of 0.6 at 540 nm. Cells were induced by incubation with 0.1 mM IPTG at 37°C/2 h. The cells were then harvested by centrifugation, washed in ice-cold 50 mM Tris HCl-buffer (pH 7.5), and suspended in extraction buffer (50 mM Tris HCl-buffer (pH 7.5), 150 mM NaCl, 10 mM MgCl_2_, 5 mM B-mercaptoethanol, 3 M guanidinium chloride, and 2 M urea). After disruption by sonication, the crude extract was clarified by centrifugation at 30,000 × g for 30 min. The rLmxMBA was expressed as a fusion protein with an N-terminus six-histidine–residue affinity tag and was purified, under denatured conditions by affinity chromatography using Ni-agarose resin (Qiagen, Hilden, DE) according to the manufacturer instructions. The collected protein was dialyzed for 48 h at 4°C against PBS.

### 2.7. Anti-rLmxMBA Antibodies

The recombinant protein LmxMBA (rLmxMBA) was obtained as described above, and female BALB/c mice were immunized with 10 *μ*g/dose. Animals received only two intraperitoneal (i.p.) doses with 15 days of interval; the immunization doses were administrated in TiterMax adjuvant (1 : 1 mixture) (Sigma, St. Louis, MO, USA), and 30 days after the immunization scheme, mice were bled by cardiac puncture to obtain immune sera.

#### 2.7.1. Expression of LmxMBA Antigen *In Vitro*

Mammalian cells were transfected with pVAX1::LmxMBA, using the X-tremeGENE HP Transfection Reagent (Roche Diagnostics, Penzberg, DE), according to the manufacturer's instructions. The HeLa transfected cells were harvested 72 h later and washed three times with PBS. Total RNA was extracted with the TRIzol reagent (Invitrogen, Carlsbad, CA, USA). Complementary DNA (cDNA) was derived from RNA with the SuperScript™ First-Strand Synthesis System (Invitrogen, Carlsbad, CA, USA). LmxMBA cDNA was amplified by PCR using specific primers LmxMBA/F and LmxMBA/R, as described above. The amplified products were analyzed on a 1% agarose gel. In addition, the expression of LmxMBA was evaluated by immunoblotting of transfected cells. Glyceraldehyde-3-phosphate dehydrogenase (GAPDH) was used as a housekeeping control gene. The primers used were GAPDH/F (5′-GGTGCTAAGCAGTTGGTGGT-3′) and GAPDH/R (5′-GAGTCAACGGATTTGGTCGT-3′).

### 2.8. SDS-PAGE and Immunoblotting

Proteins were resolved on 12% SDS-PAGE and either visualized by Coomassie blue or electrophoretically transferred onto a nitrocellulose membrane for immunoblotting. Anti-histidine antibodies at 1 : 1000 dilution and anti-rLmxMBA antibodies at 1 : 200 dilution were used as primary antibodies in TBST (150 mM NaCl, 0.5% Tween 20, 2% skim milk, and 10 mM Tris HCl pH 7.4). Bound antibodies were detected using alkaline phosphatase-conjugated goat anti-mouse IgG (Zymed Labs, San Francisco, CA) diluted at 1 : 5000, and the blot was developed with NBT and BCIP (Invitrogen, Carlsbad, CA, USA).

### 2.9. Immunofluorescence Microscopy

*L. mexicana* promastigotes and amastigotes were washed 3 times in PBS and fixated in 4% paraformaldehyde (PFA) in filtered PBS pH 7.4 for 15 min at room temperature (RT). Following an additional PBS wash, the cells were resuspended in filtered PBS and then left to sediment and adhere to the surfaces glass slides for 30 min at RT. The fixed cells were permeabilized with cold methanol for 5 min and then labeled with mouse polyclonal anti-LmxMBA diluted 1 : 100 in PBS. A secondary FITC-conjugated goat anti-mouse IgG antibody was used for fluorescent detection of the LmxMBA protein. Slides were mounted with Vectashield and DAPI (4′,6′-diamino-2-phenylindole).

### 2.10. Mouse Immunization and Experimental Infection

Four- to six-week-old female BALB/c mice (30 in total) were immunized intramuscularly. Plasmids were prepared as described and then dissolved in sterile PBS (pH 7.4) as DNA vaccines for immunization. Mice were divided randomly into three different groups of ten animals per group: one group was vaccinated with 100 *μ*g of pVAX1::LmxMBA plasmid/per mice, and as controls, one group was vaccinated with the empty plasmid, pVAX1, and another was not immunized (PBS). A three-dose immunization scheme at 15-day intervals followed by parasite challenge was adopted.

Two weeks after the last immunization, the mice of each group were challenged at the right footpad subcutaneously. Cultured promastigotes were collected, centrifuged at 350 g/5 min, washed three times with PBS, and counted in a Neubauer chamber. The inoculum was prepared in a total volume of 25 *μ*l of PBS containing 5 × 10^6^ promastigotes. The lesion size was determined weekly by measuring the thickness of the infected and healthy footpads, using a Vernier caliper, and calculating the size difference between the paw pads. The animals were sacrificed 105 days after infection, and the hind legs were amputated at the level of the subtalar joint to obtain skin tissue from the footpad. Mice were bled by cardiac puncture, and the spleens were collected for immunological purposes.

### 2.11. Determination of the Weight of Lesions and Parasite Burden

The parasite burden was quantified in skin tissue by limiting dilution culture [27]. Briefly, hind paws (infected and healthy) were removed aseptically and submerged in 3% ionized alcohol for up to 3 minutes to allow decontamination. The paws were weighed individually, and the difference between the infected and healthy paws of each mouse was taken as the net weight of the lesion. Skin sections of the footpad were obtained (approximately 20 mg), macerated in Eppendorf tubes with 1 ml of RPMI 1640 medium culture (medium containing 10% heat-inactivated SFB, 100 IU/ml penicillin, and 0.1 mg/ml streptomycin), and diluted with the same medium at a final concentration of 1 mg/ml. The material was tenfold serially diluted in RPMI 1640 medium supplemented with 10% FBS and cultured into 96-well flat-bottom plates, in duplicate. The plates were incubated at 26°C and observed under an inverted microscope (Nikon Diaphot, Nikon, Japan) every 3 days, up to a maximum of 20 days, to record the dilutions containing promastigotes. The presence of mobile promastigotes was recorded at day 20 in each well, and the concentration of parasites per milligram of tissue was calculated as the reciprocal of the highest dilution that was positive for parasites. The total parasite load of the lesion was calculated with reference to the weight of the lesion. Each positive result is expressed as the log10 number of promastigotes on the graph.

### 2.12. Cell Proliferation Assay

Mouse splenocytes were prepared as follows [28]. Spleens were taken from immunized/challenge mice and control animals under sterile conditions, dissected, and resuspended in sterile, cold PBS (pH 7.4) containing FBS 2%. RBCs were disrupted with lysis buffer, and the single-cell suspensions were adjusted to 2 × 10^6^ cells/ml with RPMI 1640 (HyClone, Logan, UT) containing 10% FBS. The diluted cell suspensions (100 *μ*l/well) were dispensed into 96-well flat-bottom culture plates (Thermo Fisher Scientific, Rochester, NY) and restimulated with 10 *μ*g/ml of rLmxMBA protein. Concanavalin A (2 *μ*g/ml), unstimulated wells, and complete culture medium were used as positive, negative, and blank controls, respectively. Each splenocyte sample was plated in triplicate. The proliferative response was measured by WST-1 (Roche Diagnostics, Mannheim, DE) according to fabricant instruction. After 72 h of culture, 10 *μ*l of WST-1 was added to each well and incubation continued for 4 h at 37°C and 5% CO_2_. Following incubation, plates were read at 450 nm. The stimulation index (SI) was calculated as the ratio of average OD value of wells containing antigen-stimulated cells to average OD of wells containing only cells with the medium.

### 2.13. Histological Analysis

Slices taken from the skin of the footpad were rinsed with PBS and fixed with 10% formaldehyde for 24 h. Fixed sections were embedded in paraffin, sectioned (6 *μ*m), and stained with hematoxylin-eosin. The presence of amastigotes and histopathological alterations were examined under a light microscope (Nikon Eclipse 80i, Nikon, Japan). Semiquantification of parasites per field was expressed in ranges of parasites observed in 10 consecutive fields of the microscope under the 100x objective. At least 100 microscopic fields were examined before a sample was reported as negative.

### 2.14. Isotyping of Antibodies

The IgG1, IgG2a, IgG2b, IgG3, IgA, and IgM immunoglobulins from DNA-vaccinated mice were evaluated by indirect ELISA assays. Blood samples were collected (*n* = 10 animals/group) from the immunized mice 105 days after the infection. The sample was centrifuged for serum preparation. The 96-well microplates (Thermo Fisher Scientific, Rochester, NY) were coated with rLmxMBA (2 *μ*g/ml) in 100 mM carbonate-bicarbonate buffer (pH 9.6) and incubated overnight at 4°C. Plates were washed with PBS-0.05% Tween 20 (PBST) and incubated for 2 h at 37°C with blocking solution (PBS containing 5% skim milk). After washing with PBST, diluted serum samples (1 : 100) in PBST were added to each well and incubated at 37°C for 1 h and washed 3 times after the reaction. As a negative control, a pool of preimmune sera was used in all experiments. For isotyping, the plates were incubated with peroxidase-labeled goat anti-mouse isotype antibodies (Abcam, Cambridge, UK) at 1 : 10000 dilution in PBS-T for 2 h at RT and then washed 4 times with PBST and PBS. The peroxidase activity was developed by 3-ethylbenzthiazoline-6-sulfonic acid (ABTS) substrate, and the optical density (OD) was read at 405 nm in an ELISA microplate reader (LabSystem Multiskan MS).

### 2.15. Statistical Analysis

The results were expressed as means ± standard errors of the mean (SEM). All data in the study were compared using the Statistical Package for the Social Sciences (SPSS) 23.0 Data Editor (SPSS Inc., Chicago, Illinois, USA). Statistical analysis was performed using one-way analysis of variance (ANOVA) followed by a Tukey post hoc test to identify significantly different groups. Differences were considered statistically significant when the *p* value was <0.05.

## 3. Results

### 3.1. Bioinformatic Analysis

**Figure 1:**
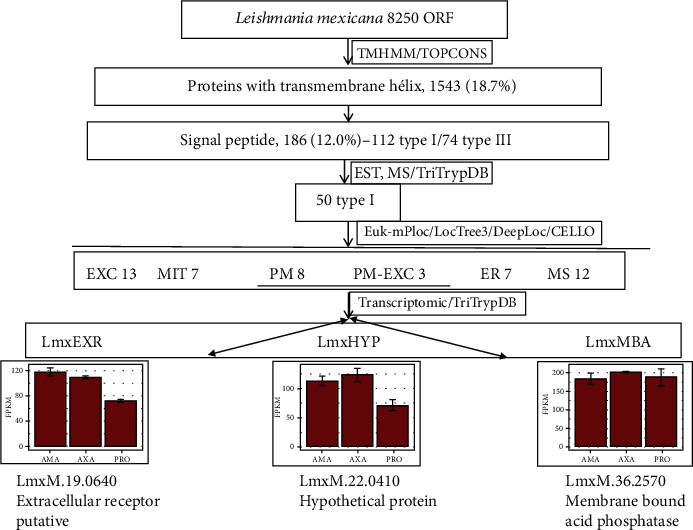
Strategy to identify putative vaccine candidates in *L. mexicana*. Abbreviations: EXC: excreted; MIT: mitochondria; PM: plasma membrane; ER: endoplasmic reticulum; MS: multiple sites.

We carried out a bioinformatic analysis of the *L. mexicana* ORF database to identify new proteins as potential candidates for the development of a vaccine. The main criterion was to select plasma membrane proteins. Analysis of the 8250 ORFs by using TMHMM [29] and TOPCONS [30] showed that 1543 proteins had transmembrane helices, and of the latter, 186 had the signal peptide. These membrane proteins were type I (112 proteins) and type III (74 proteins). Examination of the EST and MS/TriTrypDB databases indicated that 50 type I transmembrane proteins were expressed, and they presented the following subcellular location: 13 excreted, 7 in mitochondria, 8 in plasma membrane, 3 excreted/plasma membrane, 7 in endoplasmic reticulum, and 12 in various locations [31–34]. The transcriptomic analysis of the plasma membrane and plasma membrane-excreted proteins showed that the membrane-bound acid phosphatase is expressed at the same level in axenic amastigotes, macrophage-derived amastigotes, and promastigotes as compared with other proteins (Figure 1).

### 3.2. LmxMBA Localization in *Leishmania mexicana*

**Figure 2:**
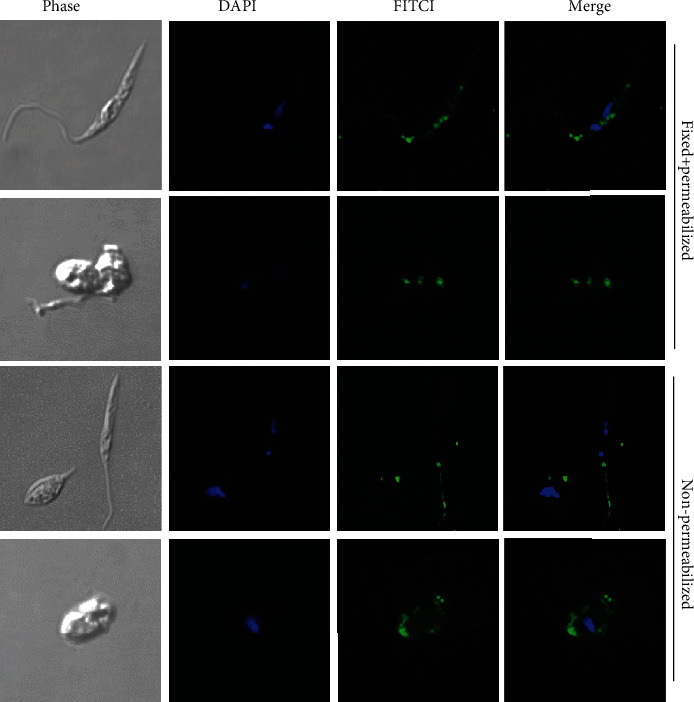
Subcellular localization of LmxMBA protein in *L. mexicana* promastigotes and amastigotes. Immunofluorescence microscopy utilizing serum anti-LmxMBA and secondary antibody coupled to FITC; nucleus and kinetoplast were marked with DAPI.

The subcellular location of LmxMBA was investigated in promastigotes and amastigotes by indirect immunofluorescence. Permeabilized and nonpermeabilized cells were incubated with the polyclonal anti-LmxMBA antibody, followed by a secondary antibody conjugated to FITC (green). As shown in Figure 2, green fluorescence was associated with the base of the flagellum in both permeabilized and nonpermeabilized promastigotes. Permeabilized promastigotes also showed green fluorescence between the nucleus and the kinetoplast. In amastigotes, green fluorescence was associated mainly towards one end of the parasite membrane.

### 3.3. LmxMBA Expression in Mammalian Cells

**Figure 3:**
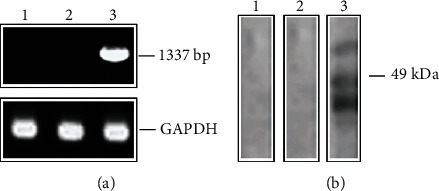
Expression of LmxMBA in transfected human HeLa cells. Nontransfected HeLa cells (1) and transfected with pVAX1 (2) or with pVAX1::LmxMBA (3) were assayed by RT-PCR (a) and immunoblotting (b) for the expression of LmxMBA 48 h posttransfection.

The recombinant plasmid pVAX1::LmxMBA was constructed to investigate the potential of the LmxMBA gene as a prophylactic DNA vaccine, in response to cutaneous leishmaniasis in the murine model. To ensure that LmxMBA could indeed be transcribed in mammalian cells, the presence of specific mRNA was established by RT-PCR from total RNA isolated from HeLa cells transfected with plasmid encoding the LmxMBA protein. RT-PCR showed amplification products at 400 bp for GAPDH in all the groups of cells (Figure 3(a)), while only the pVAX1::LmxMBA transfected cells showed amplification of the LmxMBA mRNA at 1337 bp, not so in untransfected cells or transfected with the empty vector (Figure 3(a)). In addition, the LmxMBA protein was detected in lysates of HeLa cells transfected with the plasmid encoding that protein by western blotting analysis (Figure 3(b)). The expected MW for LmxMBA protein is 49 kDa, and possibly, the lower and higher bands are degradation product and protein aggregate of the same protein, respectively.

### 3.4. Effect of DNA Vaccination on Lesion Development

**Figure 4:**
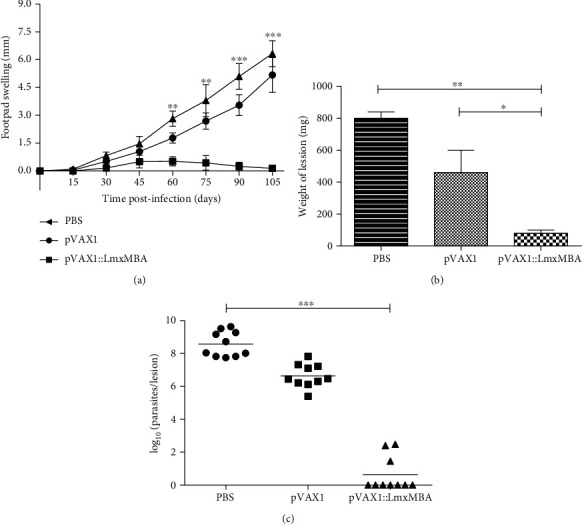
Footpad swelling (a), weight of lesions (b), and parasite burden (c) in BALB/c mice immunized and challenged with promastigotes of *L. mexicana*. Mice were immunized three times (intervals of 15 days between doses) with pVAX1::LmxMBA, pVAX1, or PBS. The results are expressed as means ± standard errors of the mean (SEM) of ten mice per group (a). The weight of the lesion was determined by amputation of the paw 105 days after the infection; the difference between the infected and healthy paws was taken as the net weight of the lesion (b). The number of parasites in the lesion was determined by limiting dilution culture, and the results are expressed as log_10_ (c). ^∗^*p* < 0.05.

Weekly measurements followed lesion development, and the lesion size kinetics showed that infection caused a progressive increase in footpad swelling in all mice from control groups. Mice immunized with plasmid encoding LmxMBA gene showed a delayed increase in lesion diameter compared to control mice, immunized with PBS (*p* < 0.0001) or pVAX1 (*p* < 0.05). In addition, statistical significance in the size of the lesion was found from 60 to 105 days after infection compared to the group immunized with PBS (*p* < 0.0001) and with pVAX1 (*p* < 0.05). Vaccination of mice with the empty plasmid pVAX1 showed continuous growth of the lesion at a slower rate than that observed in mice immunized with PBS (*p* < 0.05) (Figure 4(a)).

### 3.5. Determination of Parasite Load

There was a good correlation between lesion sizes and parasite loads. A significantly lesser amount of parasites was observed in footpad lesions of mice vaccinated with pVAX1::LmxMBA plasmid, compared to the control groups inoculated with the empty vector (*p* < 0.001) or PBS (*p* < 0.0001). The footpad of mice immunized with the recombinant plasmid also showed lower lesion weights at 105 days compared to control mice immunized with PBS (*p* < 0.01) and compared to the group immunized with pVAX1. No statistically significant differences were found (*p* > 0.05) (Figures 4(b) and 4(c)).

### 3.6. Histopathological Study

**Figure 5:**
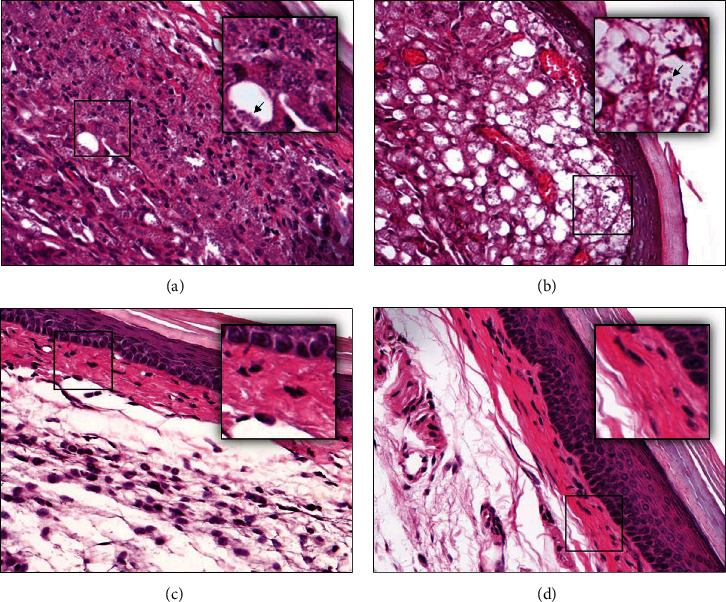
Histopathological analysis of the footpad of mice immunized with pVAX1::LmxMBA and challenged with promastigotes of *L. mexicana*. Tissues were collected 105 days after infection, and the sections were stained with hematoxylin and eosin. Vacuolated macrophages containing amastigotes were abundant in footpad lesions of mice immunized with PBS (b), as compared with mice immunized with the empty vector, pVAX1 (a); the dermis of control animals showed a massive infiltration with parasitized macrophages (arrows). In contrast, mice immunized with pVAX1::LmxMBA displayed more preserved skin with lymphocytes and macrophages containing few or no parasites (c). Microarchitecture of the dermis in mice immunized with pVAX1::LmxMBA was globally preserved in a similar manner to the healthy footpad (d).

Histopathological study showed a lesser amount of parasites in the tissue skin of mice immunized with the plasmid pVAX1::LmxMBA (Figure 5). 80% of the animals were negative in the microscopic examination of amastigotes, and the remaining 20% showed few parasites (0-20 per field) in the dermis. Mice immunized with PBS or with the empty plasmid, pVAX1, showed a high number of amastigotes (>200 per field) at the site of inoculation (Figures 5(a) and 5(b)). Besides, an infiltrate of inflammatory cells occupying the dermis and hypodermis with the presence of multiparasitic vacuolated macrophages was observed. A disorganized dermal region was also observed, with loss of normal morphology, filled by an intense infiltrate, rich in polymorphonuclear and mononuclear cells (Figures 5(a) and 5(b)). Mice immunized with the empty plasmid pVAX1 showed fewer vacuolated macrophages compared to nonimmunized mice. However, highly parasitized nonvacuolated macrophages were observed in mice immunized with the empty vector.

In contrast, immunized mice with pVAX1::LmxMBA plasmid showed a lower amount of parasitic structures per macrophage at the site of the lesion. The lesion of these animals showed a lower frequency of parasitized macrophages (20%) or total absence of them (80%) (Figure 5(c)). Vaccination with pVAX1::LmxMBA plasmid showed the typical histological characteristic in the infected footpad; a dermis with abundant collagen fibers was observed by microscopic examination, similar to control tissue (Figure 5(d)). Although there was still a mild inflammatory infiltrate in the dermis of some animals (30%) (including those with a low number of parasites per field), no damage or loss of the tissue microarchitecture was observed.

### 3.7. Lymphocyte Proliferation Assay

**Figure 6:**
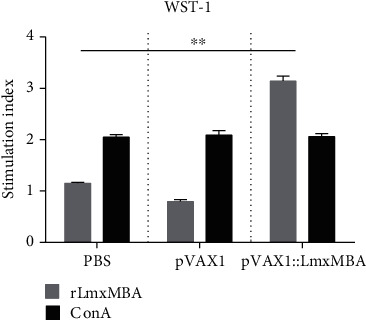
Proliferation of mononuclear cells in the spleen of mice immunized with PBS, pVAX1, or pVAX1::LmxMBA after stimulation with rLmxMBA. The mean stimulation index (SI) of vaccinated mice (injected with pVAX1::LmxMBA) was nearly 3 times greater than the mean SI of control mice (injected with PBS or pVAX1). ConA: concanavalin A. ^∗^*p* < 0.05.

To further investigate the cellular immune response elicited by immunization with pVAX1::LmxMBA, cellular proliferation assays were performed. As shown in Figure 6, mice immunized with pVAX1::LmxMBA induced a greater antigen-specific cell proliferation response, in contrast to the control groups (*p* < 0.05). This showed the ability of LmxMBA to induce spleen cell proliferation as a maker of memory T cell immune responses whereas no significant difference was observed between pVAX1 and PBS groups (*p* > 0.05).

### 3.8. Isotyping of Antibodies

**Figure 7:**
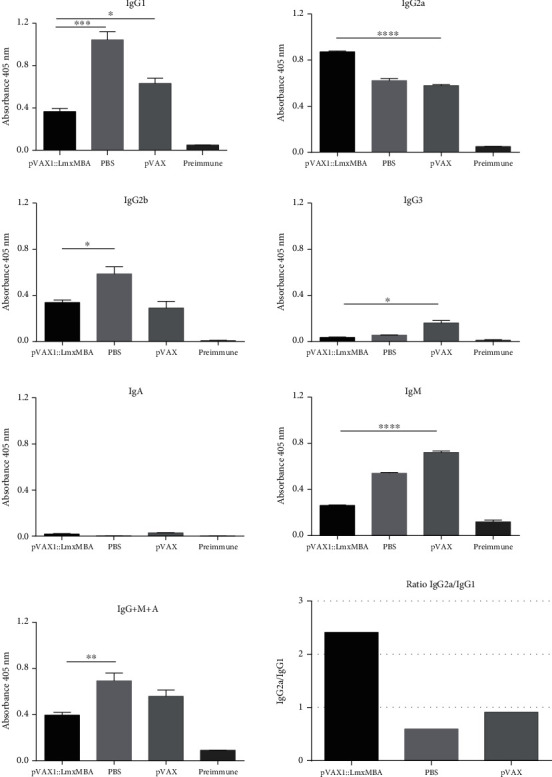
Vaccine-specific serological responses. Serum samples were collected by cardiac puncture on day 105 post infection. Plates were coated with 2 *μ*g/ml of rLmxMBA, and serum samples were diluted 1 : 100. Each bar represents the average ± standard deviation of optical densities. ^∗^*p* < 0.05.

In order to investigate the phenotype (Th1/Th2 pattern) of immune response elicited by immunization of pVAX1::LmxMBA, the isotype of antibodies was analyzed by an indirect ELISA method. As shown in Figure 7, anti-LmxMBA IgG+M+A values were significantly lower than the PBS control group (*p* < 0.01), and there was no statistically significant difference with the pVAX1 control group (*p* > 0.05). When comparing the immunoglobulin isotype levels from different groups of mice, we observed a low level of IgG1 in animals immunized with pVAX1::LmxMBA and the highest level in the control group immunized with PBS (*p* < 0.001) and pVAX1 (*p* < 0.05). On the other hand, a statistically significant difference in IgG2a levels was detected between mice immunized with pVAX1::LmxMBA and control mice, pVAX1 (*p* < 0.001) or PBS (*p* < 0.001). In all groups of mice, the levels of IgG3 and IgA remained low. The IgG2a/IgG1 ratio was >1 and higher in immunized mice with pVAX1::LmxMBA compared to control mice, PBS (*p* < 0.05) or pVAX1 (*p* < 0.05). This ratio does not differ significantly between mice immunized with PBS and pVAX1 (*p* > 0.05) (Figure 7).

## 4. Discussion

The control of leishmaniasis through vaccination is an increasing challenge. Although several attenuated and protein-based vaccines have protected against different species of *Leishmania* in different animal models, some of them have intrinsic disadvantages, such as the risk of reversion to virulence and the selection and use of adjuvants, respectively [8, 10, 34]. Vaccination with plasmid DNA is an active area of research applied to cancer and microbial pathogens associated with intracellular infections [19, 35]. DNA vaccines have been reported to persist in skeletal muscle for at least 19 months and are easy to produce in high purity [36, 37]. DNA vaccines are temperature stable (room temperature 2 to 8°C) over extended periods, enabling easier shipping and storage and allows to dispense with the cold chain used in conventional vaccines [37, 38]. Several plasmids used as DNA vaccines are stable for more than 3 years at 4°C, at least 1 year at 25°C, and 1 month at 37°C [39]. DNA vaccination has been used against different diseases due to its potential to induce both humoral and cellular immune responses and together with reverse vaccinology represents a potentially new approach to design vaccines against leishmaniasis and other infectious diseases such as hepatitis B and C, malaria, and human papillomavirus infection [38, 39].

We identified a potential candidate for a protective vaccine by reverse vaccinology. The criteria by which we chose to select potential vaccine antigen initially included a focus on surface proteins and sequence conservation among *Leishmania* species. Other considerations were that the chosen candidate must lack sequence identity with human proteins and the ability to express the protein in a high-throughput manner for vaccine production, e.g., we discard insoluble proteins with more than two transmembrane helices. In this regard, the LmxMBA protein has a 26.9% identity with a human protein. However, importantly, it has no shared stretches greater than six amino acids and is therefore unlikely to contain any MHC I or II epitopes. Therefore, this protein is expected to have no safety concerns if administered during vaccine trials.

Infection with *L. mexicana* causes many CL cases in the Americas and, less frequently, diffuse cutaneous and mucocutaneous leishmaniasis, even more disfiguring forms of the disease. *L. major* can also cause CL in the old world, and on the other hand, the deadliest form of the disease, VL, is mainly caused by *L. donovani* and *L. infantum* [40–42]. Thus, when the selected antigen is conserved in the various *Leishmania* species, it is possible that a vaccine directed against CL could protect against these other forms of leishmaniasis. In this way, the candidate vaccine could confer cross-protection against other *Leishmania* species because the extramembrane region of the LmxMBA protein has >90% identity with *L. major*, *L. donovani*, and *L. infantum* homologues. Even when considering the most distant species that belong to the alternated subgenus Viannia, the candidate antigen exhibits at least 71% sequence conservation with *L. braziliensis*, *L. panamensis*, and *L. guyanensis* species.

LmxMBA is a protein exposed on the membrane of *L. mexicana* and belongs to the acid phosphatase expressed in the flagellar pocket in promastigotes and amastigotes. The LmxMBA gene [43] and its homologues in *Trypanosoma brucei* [44] encode proteins that have been observed in small vesicle-like structures close to the flagellar pocket, and protein overexpression in *L. mexicana* promastigotes leads to the translocation of the protein to the cell surface and the flagellar pocket membrane.

In this study, we found the LmxMBA protein is located mainly in the flagellar pocket membrane of wild-type promastigotes and amastigotes. It is less frequent on the surface or in organelles of the parasite, including the flagellum. Proteins exposed on the plasma membrane have been shown to represent the most accessible pool of potential vaccine targets for the development of vaccines. Therefore, LmxMBA as a membrane-bound protein may represent an ideal candidate for the development of a DNA vaccine against *L. mexicana.*

There are several key components involved in the immune response to *Leishmania* infection. The outcome of infection depends on the host's ability to mount a protective Th1 response versus the ability of the parasite to evade and manipulate the host's immune system [45]. Macrophages and effects molecules, dendritic cells, T helper cells, cytotoxic T cells, natural killer cells, and cytokines are all considered to play essential roles in the immune response to *Leishmania* infection [45]. In this study, DNA vaccine encoding LmxMBA of *L. mexicana* was able to promote humoral and cellular immune responses, reducing the clinical manifestations of the cutaneous disease in genetically susceptible mice. Most animals (70%) did not show any footpad swelling at 105 days after challenge, the parasites were cleared from the injection site, and the dermis was free of inflammatory inflates. The rest of the animals in this group (30%) showed minimal and transient swelling at the inoculation site and a low number of parasites and mild inflammatory infiltrates. A delay in the development of the disease was observed in all animals immunized with the LmxMBA gene. In cutaneous infection with *L. major*, vaccination with the divergent part of H2B protein confers clinical and parasitological protection against a virulent challenge, considered a protective effect when the size of the lesion and the parasite load in the vaccinated mice were significantly smaller than that observed in control animals [46]. In the experimental mouse model of visceral leishmaniasis caused by *L. donovani*, Melby et al. [47] showed that DNA vaccination using a cDNA library, containing notably H2B, partially protects BALB/c mice against a virulent challenge with *L. donovani*. The protective effect induced by the vaccine was partial since, during infection in this experimental model, vaccination was more efficient at clearing parasites from the liver than the spleen.

Immunization with the empty plasmid generated macroscopical lesions in all of this group's animals (100%). However, the size was smaller than the PBS control group's lesions, the parasite load remained high, and there was tissue damage with chronic inflammatory infiltrates. This apparent protective effect may be due to the CpG motif. The empty vector has CpG motifs that act as a “danger signal” and as an enhancer of the Th1 immune response in DNA vaccination through interaction with TLR9-positive cells, and this could be activating a nonspecific immune response against infection [24, 25], thus inducing a lower swelling without reducing the parasite load.

As expected, parasites in the lesion were evident in all the animals in the control groups by staining tissue. However, in the group of animals immunized with the candidate antigen, only 20% showed parasites by stained skin and 10% more by limiting dilution culture; this is consistent with other studies in mice since limiting dilution culture allows quantification of parasite burdens at least 50- to 100-fold lower than the minimal parasite burdens quantifiable on stained organs [48]. Due to its high sensitivity, this method is particularly suitable for experimental situations in which parasites cannot be visualized in stained organs: e.g., after therapeutic intervention or a low parasite persistence. It would be interesting to extend the animals' monitoring time to observe the complete elimination of the parasite in the infected tissue or the persistence. Nevertheless, an essential characteristic of leishmaniasis is that residual parasites could remain in the host despite the lesion's disappearance, probably for a very long time [49].

The vaccine also showed that the control was given by a Th1-type response, since mice immunized with pVAX1::LmxMBA produced significantly higher levels of IgG2 compared to control mice, which conversely showed significantly high levels of IgG1. Many reports show that during *Leishmania* infection, higher amounts of the IgG1 isotype in comparison with IgG2a are associated with disease progression [50, 51]. This IgG2a/IgG1 > 1 ratio is representative of a Th1-type immune response because IFN-*γ*, produced by Th1-like cells, suppresses the induction of IgG1 and increases the production of IgG2 antibodies [52]. On the other hand, levels of IgM, IgG2b, and IgG3 were significantly lower in the experimental group compared to the control mice. Humoral immune response has been evaluated in different stages of the disease in humans with leishmaniasis. Elevated levels of IgM antibodies have been found in the serum of patients with visceral leishmaniasis who are in the acute phase of the disease, IgM levels remain high in patients resistant to sodium stibogluconate (SAG), and patients who respond to SAG showed a decrease in the levels of total IgG, IgM, and IgE [53].

It has been mentioned that the deletion mutant for this gene is able to infect BALB/c mice and peritoneal macrophages, thus suggesting that LmxMBA is neither involved in the infection process nor required for amastigote survival in the infected host cell [54]. It could be possible that the immune response induced by DNA vaccination with the LmxMBA gene affects the activity of LmxMBA or homologous proteins essential for the infection and survival of amastigotes within the host. The *L. mexicana* genome codifies for 3 membrane-bound acid phosphatases and 1 secreted histidine acid phosphatase with 53-56% homology to LmxMBA (data not shown).

In conclusion, immunization with the gene LmxMBA can induce partial protective immunity against *L. mexicana* infection in the murine model. Until now, the efficacy of the single-dose vaccine candidate has not been evaluated. Future studies would be of great interest to test our DNA vaccine under additional conditions, mainly if lower doses of the vaccine or even a single dose is sufficient to reduce the clinical characteristics of visceral and cutaneous leishmaniasis in other animal models.

Our data identify a vaccine antigen that could potentially be used in target vaccination campings in *L. mexicana*-endemic regions. Due to possible cross-reactivity against other *Leishmania* species, it would be essential to evaluate the efficacy of this DNA vaccine against the parasitic challenge with species that cause visceral leishmaniasis in large animals, including dogs and also in humans, in order to find universal vaccine leishmaniasis.

## Data Availability

The data that support the findings of this study are available from the corresponding author upon reasonable request.
